# Mechanobiology in cardiac mechanics

**DOI:** 10.1007/s12551-021-00827-4

**Published:** 2021-08-27

**Authors:** Michael Sheetz

**Affiliations:** Biochemistry and Molecular Biology, University of Texas Medical Branch Galveston, TX 77555 Galveston, USA

## Abstract

The contraction-relaxation cycle of the heart is one of the most robust mechanical systems in the body that adapts rapidly to the body’s needs by changing mechanical parameters. In many respects, we can consider the cardiac system as a complex machine and can use engineering approaches to describe its function. The classical physiology of the heart also focused on understanding function but the new molecular level tools in light microscopy and nanoengineering now enable a deeper understanding of the physiology. The field of mechanobiology has emerged with a focus on how mechanical activity alters biological systems at the molecular level and how those systems in turn control mechanical parameters. In the case of mechanical activity, there are clearly benefits of exercise for the heart, for cancer patients, and for aging but we do not understand the links at a molecular level. Why does regular exercise benefit the heart? We have some preliminary clues at a molecular level about the benefits of physical activity in the cases of cancer and aging; however, there is less known about how exercise affects cardiovascular performance. Unlike the omics approaches which generally link proteins to processes, a mechanobiological understanding of a process explains how forces and mechanical activity will regulate the process through modifications of protein activities. In other words, mechanical activity is an essential component of most biological systems that is transduced into biochemical changes in protein activity. Further, it follows logically that if a mechanical parameter of the cardiac system is typically controlled, then cellular mechanosensing systems must be able to directly or indirectly measure that parameter. The challenge is to understand how changes in activity of the heart are controlled in the short term and then how the system adapts to the integrated level of activity over the longer term. By way of introduction to molecular mechanobiology, I will present examples of mechanosensing from the molecular to the cellular scale and how they may be integrated at the cell and tissue levels. An important element of Mechanobiology at the system level is the physiological state of the cell: i.e., the cell in a senescent state, a cancer state, or a normal cell state (Sheetz 2019). The background for the mechanobiological approach is discussed in “The Cell as a Machine” (Sheetz and Yu, Cambridge Univ Press, 2018), which considers cell states and the molecular systems underlying the important cellular functions. A major challenge in mechanobiology is the understanding of the transduction of mechanical activity into changes in cell function. Of particular relevance here is the benefit of exercise to cardiac performance. This has been seen in many cases and there are a variety of factors that contribute. Further, exercise will benefit cancer patients and will reverse some of the adverse effects of aging. Exercise will cause increased cardiac activity that will be sensed by many mechanosensory systems from a molecular to a cellular level both in the heart and in the vasculature. At a molecular level in cardiac systems, proteins are able to measure stress and strain and to generate appropriate signals of the magnitude of stress and strain that can regulate the cellular contractility and other parameters. The protein sensors are generally passive systems that give a transient measure of local parameters such as the stress at cell-cell junctions during contraction and the strain of the sarcomeres during relaxation. Large stresses at the junctions can activate signaling systems that can reduce contractility or over time activate remodeling of the junctions to better support larger stresses. The proteins involved and their sensory mechanisms are not known currently; however, the mechanosensitive channel, Piezo1, has been implicated in the transduction process in the vasculature (Beech 2018). In the case of strain sensors, large stretches of titin during relaxation can unfold more titin domains that can send signals to the cell. Two different mechanisms of strain sensing are likely in titin. The titin kinase domain is activated by strain but the substrates of the kinase are not know in vivo (Linke 2018). In the backbone of titin are many Ig domains that unfold at different forces and unfolding could cause the binding of proteins that would then activate enzymatic pathways to alter the contractile cycle to give the proper level of strain (Ait-Mou et al. 2017; Granzier et al. 2014; Granzier et al. 2009). The cell-matrix adhesion protein, talin, has eleven cryptic binding sites for another adhesion protein, vinculin, that are revealed by the unfolding of domains in the talin molecule (Yao et al. 2016). Since some domains unfold at lower forces than others, small strains will preferentially unfold those domains, making the system an excellent sensor of the extent of stretch as expected for titin. Because there is an ordered array of many titin molecules, the sensing of strain can be very sensitive to small changes in sarcomere length. Needless to say, titin is only one part of the regulatory system that controls sarcomere length. As one goes more deeply into the working of the system, it is evident that many additional mechanosensory elements are involved in maintaining a functioning cardiac system.

## Active sensing of the environment

**Fig. 1:**
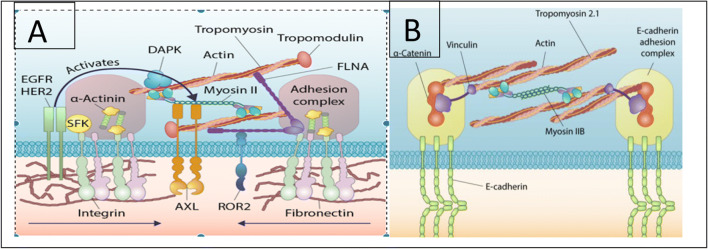
Diagrams of active rigidity sensors for **A** matrix rigidity (Wolfenson et al. 2019) and for **B** cadherin rigidity (Yang et al. 2018). In both cases, the sarcomeres contract briefly (30–60s) to a constant displacement of about 100 nm and the maximum force of contraction is proportional to the rigidity. Many downstream signaling pathways are linked to the sensors

To form the proper architecture of the heart, the cells need to sense their local environment, both the neighboring cells and the local matrix fibers. When cells die or fibers are altered, the remaining cells need to either activate repair processes or adapt to the altered conditions. In studies of fibroblasts and epithelial cells, active mechanosensing systems have been described that sense matrix rigidity (Wolfenson et al. 2016) and the rigidity of neighboring cells (Yang et al. 2018). In the case of matrix rigidity sensors, fibroblasts will assemble sarcomeric units that bridge new matrix bind sites separated by 1–3 μm and will contract to a constant length of ~100nm and relax over 30–60s (Fig. 1). At the peak of contraction, the tension in the sensor will be proportional to matrix rigidity. In fibroblasts, the rigidity sensor will activate growth on rigid matrices but will activate apoptosis on soft (Wolfenson et al. 2016; Yang et al. 2020). Similar active mechanosensors are likely to test the tension of cardiac collagen fibers and activate biomechanical processes to maintain the desired fiber tension (Pandey et al. 2018). It should be noted that these active sensors make only occasional and transient measurements of the mechanical aspects of the environment. The signals generated by the sensors are also transient but often have long-term consequences for the cell. Thus, mechanobiological processes are continuously involved in the maintenance of the proper mechanical properties of the tissue, whether it be the vasculature, the heart, or the other organs. Often changes in activity levels by individuals will be reflected in changes in organ function but those changes can take weeks to months to be fully implemented. This makes it very hard to understand the molecular-level processes that underlie those changes.

## Mechanical therapies

As it becomes clearer that mechanical activity can alter organ function through changes in cell behavior, it will become possible to use mechanical treatments to correct some undesirable behaviors. For example, we have found that ultrasound will cause apoptosis of many different tumor cells (Tijore et al. 2020), and since the ultrasound can penetrate the body, this affords the possibility of non-invasive treatment of many cancers. Exercise is a clearly beneficial mechanical therapy that may be further optimized for the organ of interest and can be combined with biochemical therapies to improve the desired outcome. There can be many benefits from the targeted use of mechanical activities to counteract certain cell behaviors and enhance others. Mechanobiology has the promise of providing many benefits to health from the non-invasive use of mechanical activity to modify cell function.

## Where to from here

As it becomes clearer that mechanical activity provides therapeutic value to the tissue and the organism, there will be an increase in molecular studies to provide a greater understanding of the molecular processes involved in transduction of stresses and strains into biochemical changes, e.g., the mechanism of stretch-induced apoptosis of tumor cells (Tijore et al. 2021). Since multiple mechanical therapies, exercise (Chen et al. 2021), stretching-massage (Zhang et al. 2019), ultrasound (Tijore et al. 2020), and potentially others, can act through similar pathways, it should be possible to design treatments for a variety of disorders that are tailored to the patient. The goal is to increase the functional activities of cells that will enable rejuvenation, repair, and regeneration without the need to resort to drugs but rather to exploit the mechanical pathways that are behind the old adage of “use it or lose it.”

## References

[CR1] Ait-Mou Y, Zhang M, Martin JL, Greaser ML, de Tombe PP (2017). Impact of titin strain on the cardiac slow force response. Prog Biophys Mol Biol.

[CR2] Beech DJ (2018). Endothelial Piezo1 channels as sensors of exercise. J Physiol.

[CR3] Chen XK, Yi ZN, Wong GT, Hasan KMM, Kwan JS, Ma AC, Chang RC (2021). Is exercise a senolytic medicine? A systematic review. Aging Cell.

[CR4] Granzier HL, Radke MH, Peng J, Westermann D, Nelson OL, Rost K, King NM, Yu Q, Tschope C, McNabb M (2009). Truncation of titin's elastic PEVK region leads to cardiomyopathy with diastolic dysfunction. Circ Res.

[CR5] Granzier HL, Hutchinson KR, Tonino P, Methawasin M, Li FW, Slater RE, Bull MM, Saripalli C, Pappas CT, Gregorio CC (2014). Deleting titin's I-band/A-band junction reveals critical roles for titin in biomechanical sensing and cardiac function. Proc Natl Acad Sci U S A.

[CR6] Linke WA (2018). Titin gene and protein functions in passive and active muscle. Annu Rev Physiol.

[CR7] Pandey P, Hawkes W, Hu J, Megone WV, Gautrot J, Anilkumar N, Zhang M, Hirvonen L, Cox S, Ehler E (2018). Cardiomyocytes sense matrix rigidity through a combination of muscle and non-muscle myosin contractions. Dev Cell.

[CR8] Sheetz M (2019). A Tale of Two States: Normal and Transformed, With and Without Rigidity Sensing. Annu Rev Cell Dev Biol.

[CR9] Tijore A, Yao M, Wang YH, Nematbakhsh Y, Hariharan A, Lim CT, Sheetz M (2020) Ultrasound-mediated mechanical forces activate selective tumor cell apoptosis. BioRxivs 2020. 10.0933/2726v1 10.1002/btm2.10737 PMC11883105 40060768

[CR10] Tijore A, Yao M, Wang YH, Hariharan A, Nematbakhsh Y, Lee Doss B, Lim CT, Sheetz M (2021). Selective killing of transformed cells by mechanical stretch. Biomaterials.

[CR11] Wolfenson H, Meacci G, Liu S, Stachowiak MR, Iskratsch T, Ghassemi S, Roca-Cusachs P, O'Shaughnessy B, Hone J, Sheetz MP (2016). Tropomyosin controls sarcomere-like contractions for rigidity sensing and suppressing growth on soft matrices. Nat Cell Biol.

[CR12] Wolfenson H, Yang B, Sheetz MP (2019). Steps in Mechanotransduction pathways that control cell morphology. Annu Rev Physiol.

[CR13] Yang Y, Nguyen E, Narayana GHNS, Heuzé M, Mège R-M, Ladoux B, Sheetz MP (2018) Local contractions regulate E-cadherin adhesions, rigidity sensing and epithelial cell sorting. bioRxiv:318642

[CR14] Yang B, Wolfenson H, Chung VY, Nakazawa N, Liu S, Hu J, Huang RY, Sheetz MP (2020). Stopping transformed cancer cell growth by rigidity sensing. Nat Mater.

[CR15] Yao M, Goult BT, Klapholz B, Hu X, Toseland CP, Guo Y, Cong P, Sheetz MP, Yan J (2016). The mechanical response of talin. Nat Commun.

[CR16] Zhang F, Gu Y, Wu L (2019). Skin-stretching device promotes the treatment effect of vacuum sealing drainage technique on phases III and IV stress-induced injuries in aged patients with chronic critical illness: a retrospective study of 70 patients. Medicine (Baltimore).

